# Response Plasticity of *Drosophila* Olfactory Sensory Neurons

**DOI:** 10.3390/ijms25137125

**Published:** 2024-06-28

**Authors:** Lorena Halty-deLeon, Venkatesh Pal Mahadevan, Eric Wiesel, Bill S. Hansson, Dieter Wicher

**Affiliations:** Max Planck Institute for Chemical Ecology, 07745 Jena, Germanyvmahadevan@ice.mpg.de (V.P.M.); ewiesel@ice.mpg.de (E.W.); hansson@ice.mpg.de (B.S.H.)

**Keywords:** *Drosophila melanogaster*, olfaction, olfactory receptor neurons, sensitization

## Abstract

In insect olfaction, sensitization refers to the amplification of a weak olfactory signal when the stimulus is repeated within a specific time window. In the vinegar fly, *Drosophila melanogaster,* this occurs already at the periphery, at the level of olfactory sensory neurons (OSNs) located in the antenna. In our study, we investigate whether sensitization is a widespread property in a set of seven types of OSNs, as well as the mechanisms involved. First, we characterize and compare the differences in spontaneous activity, response velocity and response dynamics, among the selected OSN types. These express different receptors with distinct tuning properties and behavioral relevance. Second, we show that sensitization is not a general property. Among our selected OSN types, it occurs in those responding to more general food odors, while OSNs involved in very specific detection of highly specific ecological cues like pheromones and warning signals show no sensitization. Moreover, we show that mitochondria play an active role in sensitization by contributing to the increase in intracellular Ca^2+^ upon weak receptor activation. Thus, by using a combination of single sensillum recordings (SSRs), calcium imaging and pharmacology, we widen the understanding of how the olfactory signal is processed at the periphery.

## 1. Introduction

Encoding sensory stimuli is metabolically expensive. Sensory systems have therefore evolved to minimize the cost involved, while maximizing the amount of information gained from a stimulus by adjusting to changes in the environment based on recent input history [1]. A common feature of sensory systems is the ability to adapt, i.e., to decrease the response to a constant or repeated stimulus. On the other hand, repetitive stimuli can induce sensitization, which leads to a progressive amplification of the response. A recent study states that sensitization leads to a more detailed resolution of the stimulus itself, giving a better representation of the external information [2].

The first report on sensitization was presented in a non-associative learning context on behavioral arousal, in which a novel, strong or noxious stimulus led to an increase in reflex responsiveness [3]. This phenomenon has been extensively studied in the gill-withdrawal reflex of the sea slug *Aplysia.* In this marine invertebrate, a repeatedly noxious stimulus sensitizes the siphon withdrawal reflex [4,5,6], through the presynaptic facilitation of mechanoreceptive sensory neurons [7,8,9].

Here, we focused our interest on a form of olfactory short-term sensitization described in *Drosophila melanogaster*. Sensitization in this case refers to an increased response to weak odor stimuli when repeated within a short time window (10 s–3 min) [10]. The olfactory world of insects is highly dynamic. Once emitted from the source, volatiles are dispersed and diluted in the ambient air, resulting in a filamentous plume [11]. Using a set of ∼60 odorant receptors (ORs) [12], *Drosophila melanogaster* flies are able to extract valuable information from the plume in terms of odor identity and intensity [13]. They are capable of resolving fast changes in odor pulses [14,15] and can adjust their sensitivity based on previous odor stimuli [10,16]. ORs form heteromeric complexes of an odor-specific OR (ORX) and a co-receptor termed Orco [17,18] and are expressed in olfactory sensory neurons (OSNs) housed in the antenna and maxillary palps [19]. It has been shown that different OSN types can be sensitized upon repetitive low intensity stimulation [10,20]. However, as OSNs are strongly polarized cells, different signalling mechanisms might exist across the different compartments of the neuron (i.e., soma, inner dendrite, outer dendrite). For example, sensitization at the outer dendrite of the OSN type expressing the Or22a receptor is calmodulin (CaM) dependent, but there is yet at least one more type of regulation in the other compartments that remains elusive [20]. The so far known mechanisms involved in these processes have been reviewed recently [21]. Briefly, an odor stimulus too weak to produce a robust OR activation can lead to cAMP production [22]. This messenger activates Orco, which causes Ca^2+^ influx. Orco activity can then be further enhanced via Ca^2+^-activated calmodulin (CaM) [20,23] and/or via PKC phosphorylation [24]. These processes finally result in OR sensitization.

Despite the progress made in understanding sensitization in olfaction, fundamental questions remain unanswered. Whether this regulatory process is common for all OSNs or whether it relies on the functional role of an OSN remains unknown. Regarding the mechanisms, an increase in intracellular Ca^2+^ could also be provided by intracellular stores such as mitochondria, which has been shown to play a crucial role in the olfactory response in both mammals and *Drosophila* [25,26]. However, a possible role of mitochondria in sensitization still remains elusive.

With a combination of single sensillum recording (SSR), calcium imaging and pharmacology we aim at widening the understanding of how the olfactory signal is processed at the periphery. Here, we first demonstrated differences in the response properties among the studied OSN types and we were able to show that sensitization is not a general property. Among our selected OSN types, sensitization was observed among OSNs expressing ORs tuned towards food odors and taking part in cross-fiber coding. Furthermore, calcium imaging and pharmacological experiments demonstrated that mitochondria play an active role in sensitization, contributing to the increase in intracellular Ca^2+^.

## 2. Results

### 2.1. Differential Response Characteristics between OSN Types

**Figure 1:**
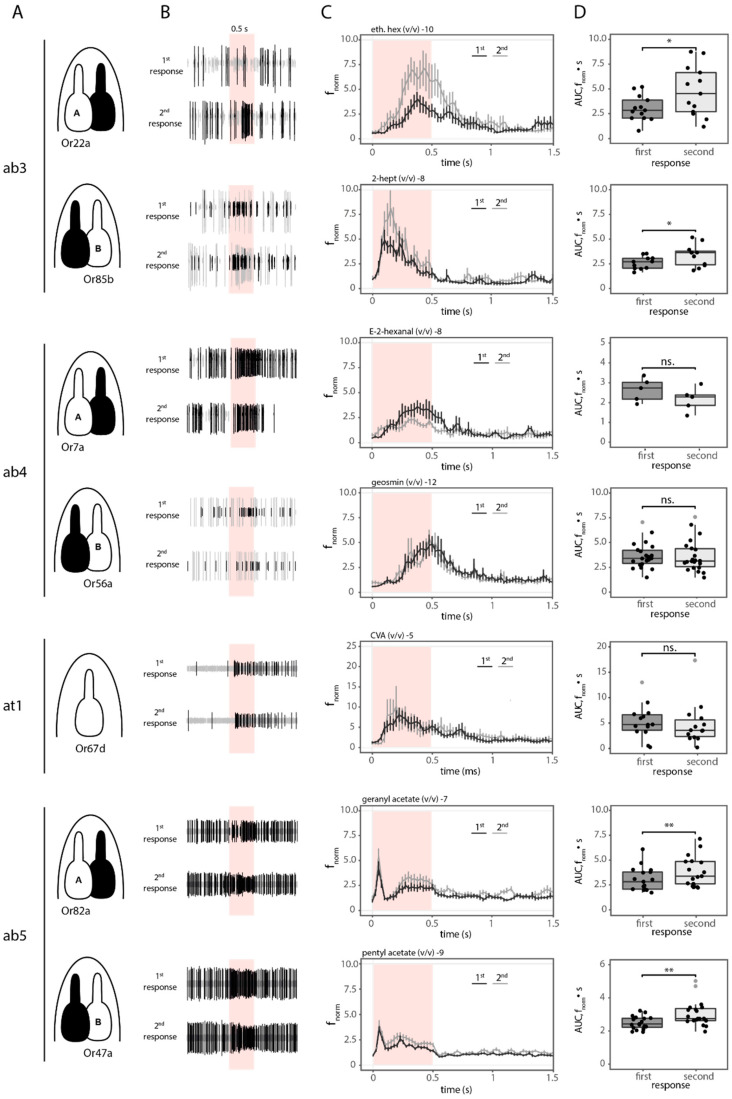
Single sensillum recordings. (**A**) Sensillum of interest (e.g., ab3) and schematic drawing of the neuron that it is being recorded from in white, partner neuron in black. (**B**) Neuronal activity of the different neurons as in (**A**) to two 0.5 s stimulation (red bar) 20 s apart. (**C**) Normalized spiking frequency (f_norm_) for first (black) and second (gray) stimulation as in (**B**). Red bar indicates odorant stimulus. Data represent mean ± SEM. (**D**) Averaged area under the curve (AUC) over 2 s corresponding to those in (**C**). Gray dots indicate outliers. Paired *t*-test: AUC_Or22a *n* = 13 pairs, AUC_Or85b *n* = 11 pairs, AUC_Or7a *n* = 5 pairs, AUC_Or56a *n* = 22 pairs, AUC_Or82a *n* = 17 pairs. Wilcoxon matched-pairs signed rank test: AUC_Or67d *n* = 14 pairs, AUC_Or47a *n* = 19 pairs. * *p* < 0.05, ** *p* < 0.01, ns not significant.

As repeated low intensity odor stimulation has been observed to lead to gradually increasing responses, we asked whether this sensitization phenomenon is a general property of OSNs. SSR enables analysis of neuronal activity [27], so we set out to investigate the occurrence of sensitization in a reduced group of OSN types by using this method. SSR data were obtained from a representative set of OSN types responding to food odors (ab3 [Or22a, Or85b], ab5 [Or82a, Or47a]), pheromones (at1 [Or67d]) and danger signals (ab4 [Or7a, Or56a]) (Figure 1A). We chose these OSN types as representatives not only because of the different behavioral significance of the stimuli they encode (food, danger, mating), but also because the receptors show different tuning properties (narrowly vs. broadly tuned). Ab4B, at1 and ab5A represent narrowly tuned receptors, whereas ab3A&B, ab4B and ab5B respond to a wide variety of odors [28]. Thus, it should be possible to find out whether such differences are related to the tuning of the receptor itself, to the meaning the odor has for the fly, or to both.

We recorded the time course, quantified the spontaneous spiking activity and the response velocity, analyzed spiking frequencies and calculated the median responses upon odor stimulation, with low concentrations of the respective best ligands. All seven neuronal types showed different dynamics (Figure 1B). We first quantified the spontaneous activity of all neurons, one second before the odor stimulation. It has been previously shown that the spontaneous activity of a neuron is determined by the OR expressed [29,30], and our results are in agreement with these observations. The spontaneous activity (spikes/s) varied significantly among the neurons (Kruskal–Wallis chi-squared = 150.38, df = 6, *p* < 0.0001; Figure S1). It is important to note that in the case of ab5, no distinction between ab5A and ab5B was possible due to the very similar spiking amplitude of both neurons. Despite the fact that the spikes were considered together, ab5A is narrowly tuned to geranyl acetate, which ensures a differential response to that of ab5B, allowing for proper separation of the neuronal spiking activity upon simulation. Partner neurons in ab3 and ab5 showed similar spiking. In contrast, ab4B had a significantly lower spontaneous activity as compared to ab4A. No differences in the spontaneous spiking activity between at1, ab3 and ab4 were found. However, probably due to the fact that the spikes were considered together, the neurons in ab5 present a significantly higher spiking rate than the others (except ab4A) (Figure S1, for detailed statistics see Table S1).

To better assess the differences in the temporal response pattern, a peri-stimulus time histogram (PSTH) was calculated [31]. Then, to accurately resolve the response kinetics, the spiking activity was normalized to 2 s before odor stimulation, providing a normalized spiking frequency (f_norm_, Figure 1C). This analysis revealed differences in the response dynamics. Ab3A, ab4A&B and at1 displayed a slow rising and longer transient phase as compared to ab3B, which showed faster dynamics. In contrast, the neurons in ab5 sensilla displayed a fast On and fast OFF response followed by a semi-plateau. The calculation of the response velocity for each OSN type (Figure S2) revealed that the second response tended to be faster in ab3A&B and ab5A. To the contrary, for ab4A&B, at1 and ab5B, the first and second responses were equally fast.

The calculation of the total area under the response kinetics curve (AUC) for each neuronal type allowed for the characterization of median responses between the first and the second odor stimulation (Figure 1D). Sensitization is observed if the second response is significantly stronger than the first to the same odor concentration [10]. We detected this phenomenon for food-odor-detecting neurons in ab3 and ab5 sensilla (Figure 1C and Table 1). In contrast, for the neurons in ab4 and at1 sensilla (responding to danger signals and pheromones, respectively) we failed to observe any sensitization (Figure 1C and Table 1). Thus, based on the physiological identity of the neurons studied, we can conclude that for our set of neurons, sensitization occurred in OSNs responding broadly to food odors irrespective of the OR tuning.

For a more extensive analysis, we set out to compare one sensitizing to one non-sensitizing neuron, morphologically and functionally. We chose Or22a as the sensitizing representative, since it is the best investigated OR [22,32,33]. Previous studies [34,35,36] have confirmed sensitization occurrence in these neurons [10,20]. Due to the ecological relevance of geosmin to fruit flies as a highly specific danger signal [37], we chose Or56a as the non-sensitizing representative.

### 2.2. Functional Differences between Or56a and Or22a Expressing OSNs

**Figure 2:**
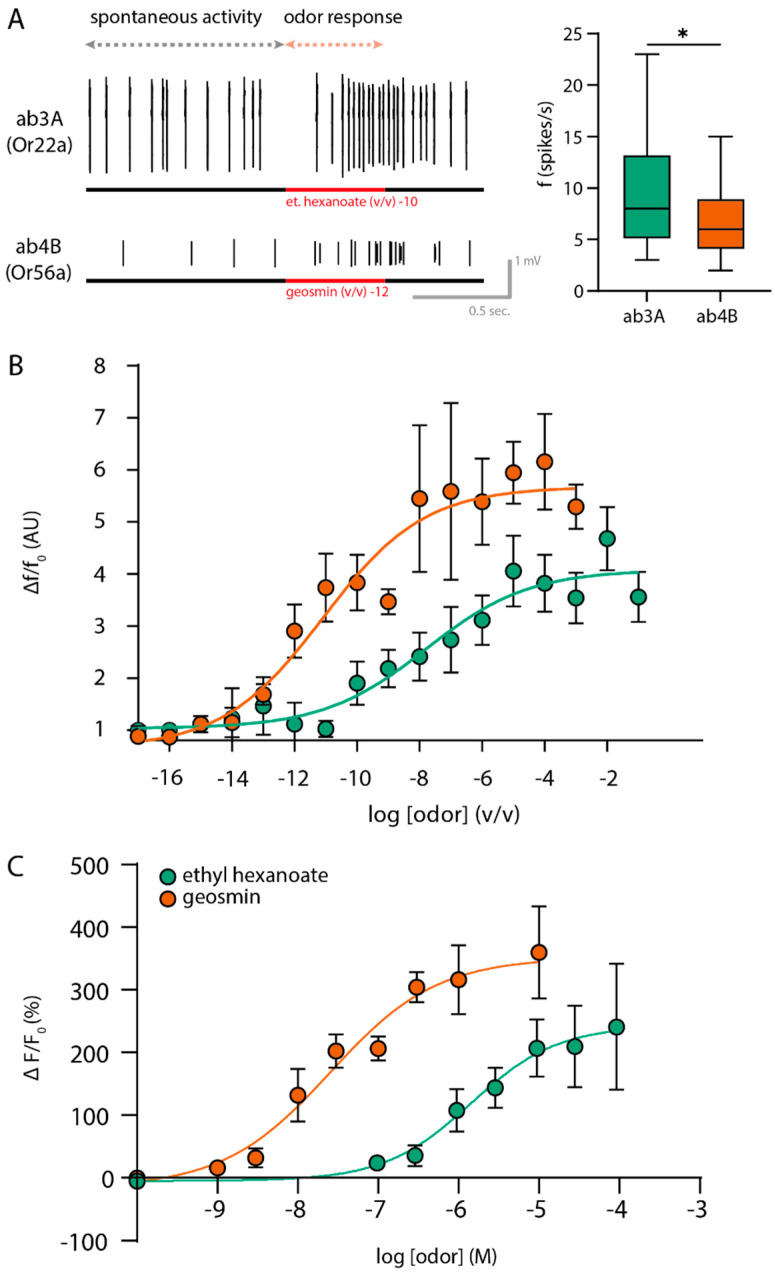
(**A**) (**Left**): Example of an SSR trace for Or22a-expressing (**top**) and Or56a-expressing (**bottom**) neurons in a 2 s time window. Spontaneous activity for 1 s is shown before the odor onset (lasting 0.5 s, indicated by the red bar). (**Right**): Spontaneous activity for 1 s is significantly lower in ab4B neurons (6.8 ± 0.6 spikes/s) compared to ab3A neurons (9.6 ± 1.1 spikes/s) (Mann–Whitney test, two-tailed, * *p* < 0.05, *n*_Or22a_ = 26, *n*_Or56a_ = 44). (**B**) SSR concentration dose–response curves for Or56a (orange, *n*_geos_ = 8) and Or22a (green, *n*_et.hex_ = 8) sensilla. Data normalized to maximal value. Curves represent sigmoidal fits described by Hill coefficient 0.29 (Or22a, eth.hex), 0.27 (Or 56a, geosmin), and logEC_50_ of −7.6 (eth.hex) and −11.1 (geosmin). Data represent mean ± SEM. (**C**) Ca^2+^ imaging concentration dose–response curves for Or22a-expressing (green, *n*_et.hex_ = 12) and Or56a-expressing (orange, *n*_geos_ = 9) neurons. The measurements were conducted in the soma. The curves represent the sigmoidal fit described by Hill for coefficient 0.8 (Or22a, eth.hex), 0.6 (Or56a, geosmin) and EC_50_ of 1.5 µM (eth.hex) and 0.025 µM (geosmin). Data represent mean ± SEM.

To characterize the functional differences between Or22a- and Or56a-expressing OSNs, we first compared the spontaneous spiking activity from the SSR measurements. Or22a displayed higher spontaneous activity compared to Or56a (Figure 2A). Secondly, to assess the OR performance, we determined the odor concentration response relationship in the SSR and calcium imaging experiments. While the dose–response curves gained from the SSR experiments directly mirror the native response of primary cells, similar data from the calcium imaging allows for the application of a highly defined concentration of odors. SSR data has been shown to be more sensitive than calcium imaging data. For calcium imaging, our concentration-dependent results for Or22a-expressing neurons were in line with those previously described [32,38]. In addition, we expanded the existing dose–response curve for geosmin, where the previously lowest tested concentration was 10^−8^ [37], by adding responses down to 10^−10^. Consistently, the SSR results and Ca^2+^ imaging dose–response experiments showed that Or56a OSNs were at least two orders of magnitude more sensitive as compared to Or22a-expressing ones (Figure 2B).

### 2.3. Morphological Differences between Or56a- and Or22a-Expressing OSNs

Recent morphometric studies have highlighted the differences in the size and general morphology among OSNs, where the “A” neuron usually is the biggest of the sensillum pair [39,40,41]. Moreover, Nava Gonzalez and coworkers also report that for basiconic sensilla, large-spike OSNs, for example ab3A, possess an enlarged inner dendrite enriched with mitochondria. Interestingly, [20] proposed intracellular Ca^2+^ sequestration as an additional mechanism contributing to sensitization in inner dendrites and the soma of Or22a-expressing neurons. In the outer dendrites, however, sensitization solely relied on calmodulin (CaM)-dependent processes. In an attempt to link these observations, we set out to determine and compare the morphology of Or56a- and Or22a-expressing OSNs.

**Figure 3:**
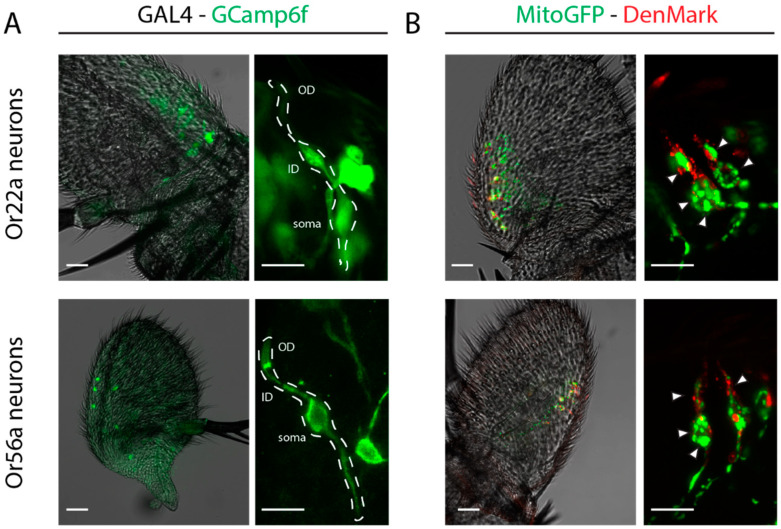
(**A**) Neuronal distribution and morphology of Gal4 lines with the fluorescent marker GCamp6f. Dotted line indicates a single neuron. White arrow indicates inner dendrite. (**B**) Mitochondrial distribution with marked mitochondria (Mito-GFP) and a dendritic marker (DenMark) under the control of the OSN Or22a- (**top**) or Or56a-Gal4 driver (**down**). White arrows indicate mitochondria. Scale bar: 20 µm for whole antenna and 10 µm for detail. OD: outer dendrites, ID: inner dendrites.

Immunostaining allowed the visualization of single OSN morphology, exposing an enlargement of inner dendrites in O22a-expressing neurons (Figure 3A, top left panel, arrowhead). This enlargement was apparently absent in Or56a-expressing neurons (Figure 3A, lower panel). In a morphological study by Shanbhag et al. in [42], and recently shown by Nava Gonzalez et al. (2021), the inner dendritic segment was described to be filled by mitochondria. To explore this possibility, we co-expressed GFP targeted to the mitochondrial matrix (Mito-GFP), together with a dendritic marker (DenMark). This allowed for the visualization of mitochondria under the control of OSN Or22a- (Figure 3B, top) or Or56a-Gal4 drivers (Figure 2B, down). The immunostaining of mitochondria confirmed that the inner dendrites of Or22a-expressing OSNs show high mitochondrial abundance.

**Figure 4:**
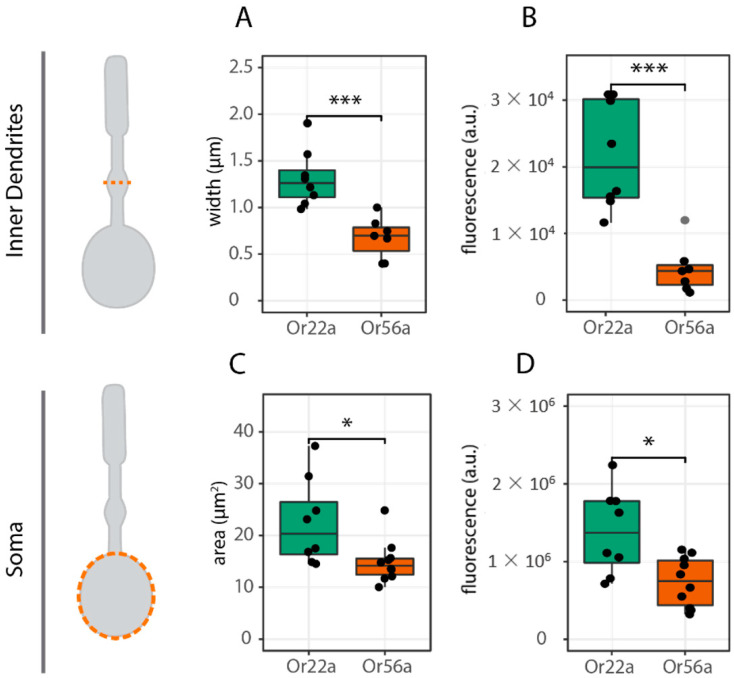
Left: Schematic drawing of neurons and the compartment analyzed, top panels for inner dendrites and lower panels for somata. Dotted orange line indicates measuring section. Middle: Width (µm) and area (µm^2^) of inner dendrites (**A**) and soma (**C**) of both neuronal populations. Left: Corrected total cellular fluorescence (CTCF) calculated as in [43], as an estimation of mitochondria abundance. There is a significant difference in the fluorescence intensity between Or22a- and Or56a-expressing neurons in both the inner dendrites (**B**) and the soma (**D**). Data represent mean ± SEM. Two-tailed *t*-test, ns not significant, *** *p* < 0.001, * *p* < 0.05. Soma: *n*_Or22a_ = 8, *n*_Or56a_ = 10; inner dendrites: *n*_Or22a_ = 8, *n*_Or56a._ = 7.

Quantification analysis of the immunostaining revealed not only that Or22a-expressing neurons have more mitochondria, but these OSNs are also larger (Figure 4). The inner dendrites of Or22a neurons are significantly enlarged (Figure 4A) and heavily packed with mitochondria (Figure 4B) as compared to Or56a-expressing neurons.

In the soma, the difference in size is not as pronounced (Figure 4C). A difference still exists in the mitochondrial abundance, being more numerous in Or22a-expressing neurons also in the soma (Figure 4D).

### 2.4. Sensitization and Mitochondria

Following these results, we wanted to evaluate whether the observed differences in mitochondria abundance could have an influence on sensitization. The involvement of mitochondria in the Drosophila olfactory response has been recently reported [26,44]. More specifically, sensitization was abolished by the interruption of mitochondrial function by the uncoupler of oxidative phosphorylation, CCCP [44]. Critical for OR function was the balance between mitochondrial Ca^2+^ uptake and release [26]. Sensitization was abolished by the induction of Ca^2+^ release via the mitochondrial permeability transition pore (mPTP), but not when previous Ca^2+^ import via the mitochondrial Ca^2+^ uniporter was blocked by Ru360 [44]. Lucke and colleagues found that auranofin, an mPTP activator [45], caused a significant reduction in the OSN response. In addition, manipulation of the mitochondrial function can influence the general sensitization of OSNs when stimulated with the Orco agonist VUAA1. The critical player in this case was also shown to be mPTP, in that the application of auranofin depressed the sensitization [44]. Therefore, we chose auranofin to evaluate the mitochondrial influence on sensitization. 

Sensitization occurs near the threshold and according to the dose responses shown in Figure 2, the chosen concentrations for the experiments were 1 nM (10^−9^) for geosmin and 0.5 µM (10^−7^) for ethyl hexanoate [20]. One of the advantages of the open antenna preparation for Ca^2+^ imaging experiments (established by [23]), is that it allows for the measuring of the receptor activity in the different compartments of the cell, mainly outer dendrites, inner dendrites and the soma. To that end, we designed an experiment consisting of double paired stimulations in an open antenna preparation. One pair stimulation under control conditions and one in the presence of auranofin were carried out. This allowed us to compare the direct involvement of mitochondria in the response of our selected cells.

#### 2.4.1. Mitochondria Are Important for Sensitization in Or22a-Expressing Neurons

**Figure 5:**
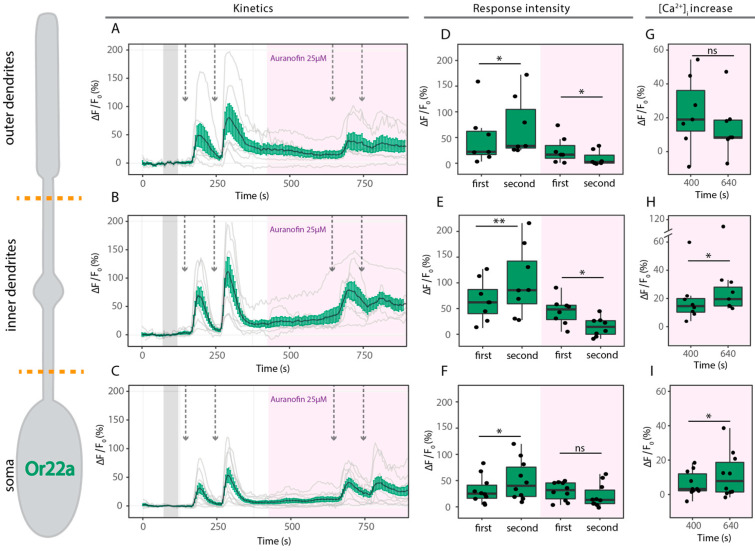
Sensitization in Or22a neurons. (**Left**): Schematic drawing of Or22a-expressing OSNs. Orange dotted lines show the division of the different cellular compartments as used for analysis. (**A**–**C**) Kinetics show averaged time course of the change in fluorescence intensity (Δ*F*/*F*_0_) in *Drosophila* OSNs after application of 0.5 µM ethyl hexanoate (arrows) in outer dendrites ((**A**), *n* = 7), inner dendrites ((**B**), *n* = 8) and soma ((**C**), *n* = 10) under control conditions (in white) and in the presence of the mPTP activator auranofin 25 µM (pink box). Gray bar indicates where data were normalized to obtain Δ*F*/*F*_0_. (**D**–**F**) Maximum increase in Δ*F*/*F*_0_ after ethyl hexanoate application in the different compartments as in (**A**–**C**). (**G**–**I**) Maximum increase in *GCaMP6f* fluorescence corresponding to [Ca^2+^]_i_ between paired stimulations in presence of auranofin 25 µM. Data represent mean ± SEM; one tail paired *t*-test, ns not significant, * *p* ≤ 0.05, ** *p* ≤ 0.01.

Sensitization was observed in all compartments of Or22a-expressing neurons under control conditions (Figure 5A–F, white box; Table 2). However, in the presence of auranofin (25 µM), the OR response was diminished and sensitization was abolished (Figure 5A–F, pink box; Table 2). Furthermore, there was an increase in intracellular calcium [Ca^2+^]_i_ in the inner dendrites and soma while in the presence of auranofin, indicating the release of Ca^2+^ from the mitochondria (Figure 5G). This result indicates that mitochondria play an active role in the sensitization properties of Or22a-expressing neurons.

#### 2.4.2. Or56a-Expressing Neurons Show No Sensitization

**Figure 6:**
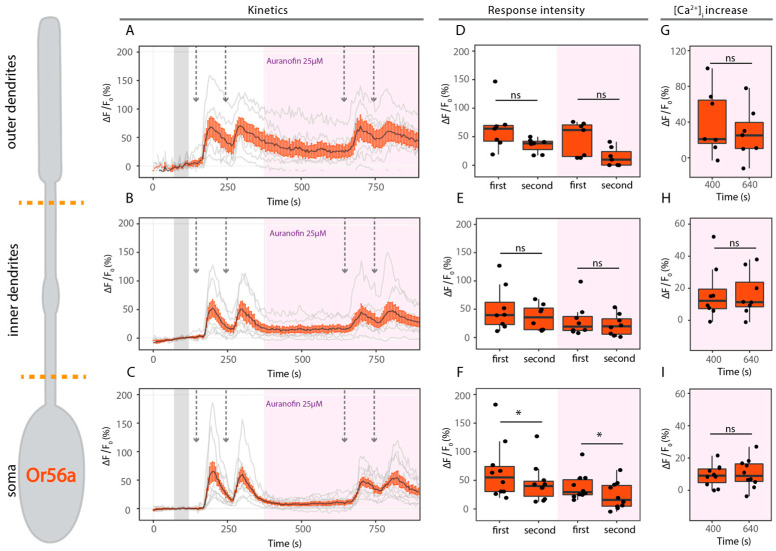
No sensitization in Or56a neurons. (**Left**): Schematic drawing of Or56a-expressing OSNs. Orange dotted lines show the division of the different cellular compartments as used for analysis. (**A**–**C**) Kinetics show averaged time course of the change in fluorescence intensity (Δ*F*/*F*_0_) in *Drosophila* OSNs after application of 1 nM geosmin (arrows) in outer dendrites ((**A**), *n* = 8), inner dendrites ((**B**), *n* = 7) and soma ((**C**), *n* = 10) under control conditions (in white) and in the presence of the mPTP activator auranofin 25 µM (pink box). Gray bar indicates where data were normalized to obtain Δ*F*/*F*_0_. (**D**–**F**) Maximum increase in Δ*F*/*F*_0_ after geosmin application in the different compartments as in (**A**–**C**). (**G**–**I**) Maximum increase in [Ca^2+^]_i_ between paired stimulations in presence of auranofin 25 µM. Data represent mean ± SEM; one tail paired *t*-test, ns not significant, * *p* ≤ 0.05.

No sensitization event was observed upon stimulation with 1 nM geosmin (Figure 6A–F white box, Table 2). Then, we wondered whether sensitization might be visible at even lower concentrations. Hence, we tested geosmin at 0.3 and 0.1 nM in the open antenna preparation. We observed responses only at 0.3 nM and, in both cases, we failed to observe sensitization (Figure S2). Finally, we performed the experiments using geosmin 1 nM, the lowest concentration used for experiments providing strong and reliable responses (Figure 2B). In the presence of auranofin (25 µM), the second response was significantly lower only in the soma (Figure 6A–F pink box, Table 2). Between the two pairs of stimulations, there was no change in [Ca^2+^]_i_ (Figure 6G). These results indicate that mitochondria are important in restoring basal calcium levels after stimulation to ensure a proper second stimulation, but play no apparent important role in controlling either the response intensity or the intracellular calcium levels.

## 3. Discussion

Understanding how odors are detected and processed at the olfactory periphery is crucial to comprehend how information is then modulated. Here, we investigated whether sensitization, an amplification of the olfactory response at the OSN level in the *Drosophila* antenna, is a widespread property in a set of OSNs of the fruit fly. In our investigation we aimed to combine findings from SSR and calcium imaging. We recognize that each method elucidates different spatial and temporal aspects of sensitization. 

Electrical responses recorded in SSR experiments happen in a time frame of milliseconds to seconds and primarily reflect ion channel activity at the OSN dendrites. Calcium imaging on the other hand, integrates ion channel activity with internal Ca^2+^ homeostasis and conformational changes of a sensor molecule. Therefore, calcium imaging yields a much more prolonged and delayed response compared to SSR. However, calcium imaging allows the recording of spatial differences in sensitization between different OSN compartments. In order to accommodate for these differences in method, we used different experimental procedures that allowed us to investigate sensitization in all its facets.

We found that OSN types expressing different ORs respond to odor stimuli with different strength and dynamics. This indicates that the tuning OrX protein not only determines the resting activity of OSNs [30], but also the characteristics of the odor response. First, and in agreement with others [30], we observed differences in the spontaneous firing of the different OSN types (Figure 2 and Figure S1). Spontaneous activity originates in the OSNs [46,47] where ORs and Orco must be functional, and sensillar components must be intact to generate a baseline firing [48]. We found that the dose-dependent responses of OSNs in calcium imaging resemble those of cells heterologous expressing olfactory receptors (with Hill coefficients of 0.61 [34]). The Hill coefficients of the dose responses from SSRs are significantly smaller (0.29/0.27) than those from calcium imaging (0.8/0.6). Even though both techniques measure the odor response of OSNs, they differ decidedly in terms of the locale and time scope. While SSR measures the instantaneous electrical responses in the outer dendrites, calcium imaging tracks changes in intracellular calcium in the soma over a long sampling window and is influenced by the cellular calcium buffering and conformational changes of the fluorescent sensor. Henceforth, it is unsurprising that either technique yields differing results. Nevertheless, the mutual <1 Hill coefficients of both the calcium imaging and SSR measurements indicate that a single odor molecule may activate multiple odorant receptor molecules. Further, the fact that cells with heterologous OR + Orco also show <1 Hill coefficients seems to imply that one odor molecule can activate multiple OR + Orco channels regardless of the presence of intracellular machinery and is rather an innate property of these channels.

Furthermore, we observed and quantified the differences in the response dynamics (Figure 1C and Figure S2). As expected for responses near the threshold, neurons in ab3, ab4 and at1 (with the exception of ab3B) showed a more tonic response [49]. In contrast, the responses of neurons in ab5 sensilla displayed a response similar to that of retinal bipolar ON cells when adapting to light [50]. These differences, observed in the response kinetics (Figure 1), are in agreement with previous studies stating that response dynamics depend on the odorant type [49], receptor type [14] and neuron identity [15]. The delayed odor response observed for ab4B and ab3A could be related to the time needed for the odor concentration to reach the neuron’s detection threshold. In the case of geosmin, with a very low vapor pressure (0.001 mmHg v [37]), it might be that only a few molecules reach the OSNs at a concentration of 10^−12^. This highlights the extreme sensitivity of these neurons, as already reported elsewhere [37]. However, to our knowledge, this is the first time such a low concentration has been used for electrophysiological recordings. 

To further investigate the response dynamics, we analyzed the maximal response velocity and time it took the neurons to reach the half maximal response velocity. We found significant differences between neuron types. Moreover, ab5A&B showed the smallest response velocity, yet their response was the least delayed. To the contrary, we found that ab4B showed fast response velocities, yet their fastest responses were delayed for a longer time and varied greatly in terms of the delay presented. Furthermore, we found a positive correlation between these analyzed metrics, indicating that OSNs might need to balance the response velocity and response delay (Figure S2).

In our sample, ab4A and ab4B represent OSNs encoding odors with negative valence and at1 for pheromone signals. Remarkably, all three failed to sensitize (Figure 1 ab4A&B and at1). Or56a-expressing neurons (ab4B) only respond to geosmin, which is produced by microorganisms, such as mold fungi [51] and bacteria [52], and has a great ecological relevance for the fly [37]. To date, geosmin is the only aversive odor found with a dedicated pathway, the other aversive odors are represented by combinatorial activation of glomeruli [37,53,54]. Its partner neuron (ab4A-Or7a) responds best to E2-hexanal and, although it has been classified as partially attractive [28], artificial activation of its target glomerulus DL5 leads to aversive behavior. Therefore, Or7a-expressing neurons can also be defined as an aversive input channel [55]. Interestingly, it has been proposed that aversive odors might be processed with a different logic than attractive ones in higher brain centers [56]. In walking flies, the detection of an odor increases the frequency of turning. After turns to aversive odors, flies moved more quickly and followed straighter paths away from the source as compared to turns following an attractive odor [57]. Such “runaway” behavior could shorten the exposure to harm. Therefore, the aversive response may rely on specific activity patterns of individual OSNs [56]. In line with this, larval OSNs carrying highly relevant information for survival are regulated differently in the AL via local interneurons (LNs). Reduced presynaptic inhibition of OSNs responding to odors associated with life-threatening situations allows *Drosophila* larvae to detect these odors less dependently of the response intensity of other OSNs [58]. Our results are in line with these observations, where the detection of aversive odors at low concentrations is sufficient to elicit a robust OSN response. Once the potential harmful situation is faithfully detected, it is likely that the source will not be further investigated, and an amplification of the signal would consequently no longer be necessary.

Along with geosmin, cVA detection is another well-established example of dedicated circuitry. Several factors contribute to high sensitivity of this pathway. First, it is detected solely by Or67d-expressing neurons in at1 sensilla targeting the DA1 glomerulus [59]. These neurons have a low detection threshold thanks to reduced spontaneous firing activity [60]. Second, the low detection threshold in combination with a high number of cVA responding neurons (∼40), renders this neuronal population highly sensitive. Third, the OSNs’ postsynaptic partners in the AL (the projection neurons (PNs)) fire a spike after only a small percentage of the OSNs have responded to a stimulus and are capable of up to 3-fold amplification [60]. As a result, PNs are able to respond rapidly to changes in the number of spikes from the OSNs [60,61]. These results, together with the fact that cVA is detected at close range, makes this neural population less susceptible to sensitization. Our results are consistent with this assumption in that, with a concentration of 0.001%, we observed clear responses, but failed to see sensitization (Figure 1 at1).

In contrast, sensitization was observed in OSNs tuned to food odors (Figure 1 ab3A&B and ab5A&B). Food odors provide crucial information about potential foraging sites, where behaviors such as mating and oviposition occur [12,28]. Or22a (ab3A) and Or85b (ab3B) represent broadly tuned receptors, responding to many odors. On the other hand, Or82a (ab5A) is narrowly tuned to few compounds, while its partner neuron ab5B houses a broadly tuned receptor (Or47a). The four OSNs project their axons into glomeruli, with positive valence in the AL (Or22a → DM2, Or85b → VM5d, Or82a → VA6, Or47a → DM3) [53]. The fact that sensitization occurs in all four OSNs indicates that this property is rather linked to the behavioral significance than to the tuning properties of the receptor. In line with this, a previous study showed that flight responses to odors eliciting attraction are dependent on the identity of the OSNs being activated [62]. Bhandawat and colleagues showed that the activation of one single neuron type was sufficient to initiate a flight surge even at low concentrations [13].

Behavioral responses to odors during flight are fast and are observed within 100 ms after the onset of OSN activity [13]. However, Getahun et al. (2013) found that OSN sensitization required an interstimulus interval of 10 s. It has been hypothesized that sensitization could aid flies following faint odor plumes when on a flying search [10,63]. So how can we reconcile these differences in time domains? Two processes might be happening in parallel, at the OSNs and at the PNs.

OSNs might be subjected to a readiness or awareness state, as described by Angioy et al. [64]. Angioy and colleagues monitored the cardiac activity of moths to evaluate olfactory detection at threshold levels. The heart response accurately indicated odor detection, but an extremely low concentration not suitable for behavioral testing. They postulate that this extreme sensitivity might be due to the formation of awareness to a certain stimulus and the readiness to respond behaviorally.

In parallel, weak OSN inputs are amplified in the PN layer as these neurons respond strongly to small increases in the OSN firing rate [61]. PNs collect information from converging the OSN input in the AL and carry the information to the higher brain center, such as the mushroom body and the lateral horn, for final processing [54]. Since PNs pool information from all OSNs expressing the same receptor in its cognate glomerulus, low odor stimuli are detected more quickly and accurately based on a single PN spike response [60,61,65]. This fast-encoding mechanism allows the animal to detect the odor onset at a very early phase [66]. In addition, as with other forms of sensitization [7,8,67], presynaptic modulation can further tune the signal transfer from OSNs to PNs [68,69]. GABA-dependent presynaptic inhibition has been reported to affect the gain control of OSNs through lateral interneurons (LNs). Interestingly, OSNs responding to CO_2_, an innate aversive cue, shows low levels of GABA receptors, which indicates reduced presynaptic inhibition [70]. This may allow for a finer detection, as seen for *Drosophila* larva [58]. It would be interesting to evaluate whether sensitizing neurons express higher levels of GABA receptors, indicating higher susceptibility to presynaptic inhibition and lateral modulation.

As a conclusion, and in agreement with others [56], we propose that odors of extreme ecological significance, such as pheromones and alarm signals, are perceived differently. For these dedicated pathways, there is an investment in more accurate detection at the OSN level; therefore, further sensitization is not needed. However, for food odors, modulation at the OSN level together with the high sensitivity of the PNs to the OSN output ensures faithful coding even at low odor concentrations.

This provides a theoretical framework on the use of sensitization for a flying insect. But what are the mechanisms that make sensitization possible in a subset of the OSN population? To answer this question, we focused on one representative example of each class: Or22a- and Or56a-expressing neurons.

Getahun et al. (2013) postulated that sensitization is intrinsic to the particular OSN type, and our results are in agreement with this observation. When we examined sensitization in their native environment, only Or22a- but not Or56a-expressing neurons were sensitized. However, when heterologously expressed in HEK cells, both Or22a and Or56a showed sensitization [20]. In addition, the study by Mukunda et al. also showed that sensitization was differently regulated in the distinct compartments of Or22a-expressing neurons, being exclusively CaM dependent only in the outer dendrites [20]. These results indicate that the receptor alone can be sensitized, but the intrinsic properties of the neuron define the final response. In the future, the heterologous expression of Or56a in an empty neuron system could yield further insight on the interplay of receptor properties and cellular architecture. Considerable effort has been spent to understand the diversity of the responses of OSNs as a function of expression of different odor receptors [14,71]. Nevertheless, it has become clear that receptors alone cannot explain the sensitivity, specificity and temporal precision observed in odor-evoked neuronal activity [72,73]. OSNs are classified according to their presence in different sensillum types, namely basiconic, trichoid and coeloconic [74], and their responses to different chemical classes [28]. Only recently, the morphological features of different OSN types have been systematically examined [39]. This study not only reveals a difference in the size between partner OSNs within a single sensillum, but also different dendritic branching and, particularly interesting, in mitochondrial abundance. These different morphological aspects of OSNs will likely influence olfactory function.

Our immunostaining results are in agreement with the morphological differences observed by others before [39,40]. Or22a-expressing neurons are bigger and have an enlarged inner dendrite packed with mitochondria as compared to Or56a-expressing neurons (Figure 3 and Figure 4). Moreover, in agreement with our hypothesis for higher sensitivity in highly specific OSNs (previous section), geosmin-detecting neurons were shown to be two orders of magnitude more sensitive than those detecting ethyl hexanoate (Figure 2). These results allowed us to design an experiment in which we could test the influence of mitochondria on sensitization in a selected neuron.

Under control conditions, the results from the Ca^2+^ imaging experiments are consistent with the SSR data, as sensitization is only observed in Or22a-expressing neurons (Figure 5 and Figure 6). The general reduction in the response intensity observed in the presence of auranofin for both neuronal populations could be due to the presence of Ca^2+^ hotspots in the vicinity of ORs. Ca^2+^ accumulation near the plasma membrane because of the activation of the mitochondrial permeability transition pore (mPTP) could lead to an early channel closure, as is known for other Ca^2+^ carrying ion channels [75]. Alternatively, a reduction in the response could be related to other auranofin effects [76]. However, for Or22a-expressing neurons an increase of the intracellular calcium concentration ([Ca^2+^]_i_) indicates auranofin-dependent activation of mPTP. In addition, as a result of auranofin application, sensitization is no longer observed (Figure 5). In *Drosophila melanogaster*, mitochondria play an active role as regulators of the odorant response in OSNs [26,44]. Furthermore, mitochondria were shown to be fundamental in shaping OSN response profiles to odors and also in maintaining sensitivity in the olfactory signal process of mammals [25]. We propose that, for Or22a-expressing neurons, Ca^2+^ release from mitochondria could contribute to further activation of Orco through feedback loops mediated by PKC or calmodulin (CaM) [21]. This would drive the OR to a sensitized state, resulting in an amplification of subsequent responses. However, upon auranofin-dependent activation of the mPTP, a slow increase in the [Ca^2+^]_i_ occurs. This indicates Ca^2+^ release, as observed before by Lucke et al., (2020). Thus, as Ca^2+^ is no longer stored in the mitochondria, there is no contribution of mitochondrial calcium to the response elicited by ethyl hexanoate stimulation and sensitization is no longer present.

In contrast, in Or56a-expressing neurons mitochondrial calcium appears to play a different role. Upon auranofin addition, there was no significant increase in the intracellular calcium concentration (Figure 6G–I). We believe that in this case, mitochondria serve mainly as a calcium clearance organelle to ensure a rapid return to basal levels. The influence of mitochondria in sensory response recovery has been previously reported in *D. melanogaster* OSNs [26] and in the photoreceptors of zebrafishes. In cone photoreceptors of zebrafish, mitochondria are tightly clustered between the outer segment and the cell body [77]. This disposition allows for mitochondrial Ca^2+^ clearance from the outer segment upon stimulation. This process is essential to promote response recovery [78]. Similarly, mitochondria present in the soma and inner dendrites serve a function for Ca^2+^ uptake after the first response to ensure a rapid return to basal levels (Figure 6B,C, white box). However, in the presence of auranofin this is no longer possible, thereby resulting in a reduction in the second response (Figure 6E,F).

## 4. Materials and Methods

### 4.1. Fly Rearing and Fly Lines

*Drosophila melanogaster* flies were reared under a 12 h light/12 h dark cycle at 25° on conventional agar medium. A list of all flies used can be found in Table 3.

### 4.2. Single Sensillum Recordings (SSRs)

A set of ORs was chosen to account for the variability in the *Drosophila* OR repertoire. Neurons in ab3 and ab5 sensilla respond to food-related odors and are broadly tuned, except for ab5A, which is narrowly tuned [28,50]. The Ab4 sensillum class accounts for aversive encoding neurons. Ab4A neurons are broadly tuned, while Ab4B are narrowly tuned to one compound: geosmin. This specific compound has a great ecological value for the fly, since its presence indicates harmful bacteria eliciting avoidance [35]. At1 houses the cVA sensing neuron, involved in social aggregation and sexual behaviors [77,78]. A summary of the target sensilla and chemicals used for the SSR can be found in Table 4.

The SSR experiments were carried out with wild-type *D. melanogaster* Canton-S (WTcs, stock #1), obtained from the Bloomington *Drosophila* Stock Center, Bloomington, IN 47405-7005, USA (www.flystocks.bio.indiana.edu accessed on 26 June 2024). A 5–8-day-old male or female fly was held immobile in a 200 μL pipette tip and fixed on a glass side with laboratory wax. The funiculus (third antennal segment) was fixed in such a position where either the medial or the posterior side faced the observer. Extracellular recordings were conducted using electrochemically (3M KOH, Merck Millipore, Darmstadt, Germany) sharpened tungsten electrodes by inserting a ground electrode in the eye and inserting a recording electrode into the base of the sensilla using a micromanipulator system (Luigs and Nuemann SM-10, Ratingen, Germany). The sensilla were visualized with 1000× magnification using a binocular microscope (Olympus BX51WI, Hamburg, Germany). The signals were amplified (Syntech Universal AC/DC Probe, Buchenbach, Germany; www.syntech.nl accessed on 26 June 2024), sampled (96,000/s) and filtered (3 kHz High–300 Hz low, 50/60 Hz suppression) using a USB-IDAC. The neuronal activity was recorded using AutoSpike software (v3.7) for 3 s pre- and 10 s post-stimulus. The stimulus was delivered for 500 ms and was added to pre-humidified air being constantly delivered to the fly at a rate of 0.6 LPM.

The stimulus was prepared by pipetting 10 μL of the desired compound dissolved in hexane (or mineral oil for ab3 sensilla) onto a filter paper with a diameter of 10 mm and placed inside a glass Pasteur pipette. No more than 3 sensilla were recorded from each fly and the odors were used once. The paired stimulations were 20 s apart, and there was a 2 min interval between the pairs.

For odor application, a stimulus controller (Stimulus Controller CS-55, Syntech, Buchenbach, Germany) was used, which produced a continuous airstream flow of 0.6 LPM air, monitored by a flowmeter (Cole-Parmer, Wertheim, Germany, www.coleparmer.com accessed on 26 June 2024). During stimulation, the airflow bypassed a complementary air stream (0.6 l/min during 0.5 s) through a stimulus pipette placed roughly 3 cm from the preparation.

### 4.3. Confocal Imaging

By crossing the fly lines 3 × 4 and 5 × 6, we generated flies with marked mitochondria (Mito-GFP in green) and a dendritic marker (DenMark [79] in red) under the control of the OSN Or22a- or Or56a-Gal4 driver, respectively. This allowed us to observe differences in the mitochondrial distribution in the dendrites of these two different OSN populations.

Images were acquired on a cLSM 880 device (Carl Zeiss, Oberkochen, Germany), using a 40× water immersion objective (C-Apochromat, NA: 1.2, Carl Zeiss), and adjusted for contrast and brightness by using the LSM Image Browser 4.0 (Carl Zeiss).

### 4.4. Antennal Preparation

For the calcium imaging experiments, antennae of 4–8-day-old females were excised and prepared as described in [23]. Briefly, the flies were anesthetized on ice, the antennae were excised and fixed in a vertical position with two-component silicone and, finally, immersed in *Drosophila* Ringer solution (in mM: HEPES, 5; NaCl, 130; KCl, 5; MgCl_2_, 2; CaCl_2_, 2; and sucrose, 36; pH = 7.3). After, the funiculus was cut allowing access to the OSNs for the experiments. Throughout the experiments, the antennae were submerged in *Drosophila* Ringer solution.

### 4.5. Calcium Imaging

A monochromator (Polychrome V, Till Photonics, Munich, Germany) coupled to an epifluorescence microscope (Axioskop FS, Zeiss, Jena, Germany) was employed for imaging. A water immersion objective (LUMPFL 60×/0.90; Olympus, Hamburg, Germany), controlled by an imaging control unit (ICU, Till Photonics) was used. Fluorescence images were acquired using a cooled CCD camera controlled by the TILL Vision 4.5 software (TILL Photonics).

GCaMP6f was exited with 475 nm of light at 0.2 Hz frequency, with an exposition time of 50 ms. Emitted light was separated by a 490 nm dichroic mirror and filtered with a 515 nm long-pass filter. The TILLVision software was used to subtract the background fluorescence and to define the regions of interest (ROI) characterized by a change in the [Ca^2+^]_i_ indicated by a change in fluorescence. The response magnitude was then calculated (∆*F*/*F*_0_) as a percentage, following [23].

The experiments lasted 15 min, with a sampling interval of 5 s. The samples were continuously perfused during the experiments with a bath solution in a perfusion/recording chamber (RC-27, Warner Instruments Inc., Hamden, CT, USA).

As sensitization occurs near the activation threshold, we checked whether the chosen stimuli were appropriate to elicit sensitization. Given that sensitization disappears within 2 min, we chose this time interval between sensitization attempts. To check if these conditions were appropriate to perform sensitization experiments in Figure 5 and Figure 6, we tested OSNs under standard conditions using the synthetic Orco agonist VUAA1 as a control (Figure S4).

OSNs were stimulated by the application of 10 µL of ethyl hexanoate (0.5 µM) or geosmin (1 nM) 2 cm away from the sample. Two control stimulations were performed 100 s apart. Then, the solution was changed into *Drosophila* Ringer + Auranofin 25 µM and 2 other paired stimulations were performed.

The free intracellular Ca^2+^ concentration ([Ca^2+^]_i_) was determined with the fluorescence ratio method and calculated as described in [23].

### 4.6. Chemicals

Auranofin (C_20_H_34_AuO_9_PS), ethyl hexanoate, (±)-Geosmin, 2-heptanone, E2-hexanal, geranyl acetate and pentyl acetate were purchased from Sigma Aldrich (Steinheim, Germany). In addition, 11-cis-Vaccenyl acetate (cVA) was purchased from Pherobank (Pherobank B.V., Wijk Bij Duurstede, The Netherlands). All chemicals have ≥97% purity.

VUAA1 (N-(4-ethylphenyl)-2-((4-ethyl-5-(3-pyridinyl)-4H-1,2,4-triazol-3-yl)thio)acetamide) was synthesized by the “Mass Spectrometry/Proteomics” group at the Max Planck Institute for Chemical Ecology (Jena, Germany).

### 4.7. Data Analysis

The SSR traces were analyzed using AutoSpike32 (Syntech NL 1998). For response kinetics (Figure 1), the spike frequency ratios were analyzed as PSTH histograms in 25 ms bins. By dividing each 25 ms frequency by the average pre-stimulus frequency over 2 s, a normalized frequency ratio (f_norm_) per each time bin was obtained. PSTH represented in the figures shows the normalized means ± SEM for *n* paired stimulations. Between 2 and 4 flies were used for each odor, and no more than 3 sensilla per fly. To obtain the response velocity, the first derivative of f_norm_ was calculated. To test for the occurrence of sensitization, the area under the curve (AUC) was calculated for all sensilla. For a comparison two-tailed paired t-test or Wilcoxon matched-pairs signed rank test was performed.

For fluorescence quantification of the mitochondria (Figure 3), ImageJ 1.54h was used following the method described by McCloy, R. A and colleagues [50]. Briefly, an outline was drawn around each soma and a transversal line through the inner dendrites to measure the area or width and mean fluorescence, along with the background. The corrected total cellular fluorescence (CTCF) = integrated density − (area of selected cell × mean fluorescence of background readings), was calculated. Two-tailed unpaired Student t tests were then performed.

The data analysis and graphs were generated using RStudio (Version 1.3.1093) and GraphPad Prism 9.

The figures were customized with Adobe Illustrator CS5 (Version 15).

## 5. Conclusions

Evidence that OSNs have a greater role than previously considered in the processing of olfactory signals is growing [68,74]. We expand this knowledge by showing that the first modulation of the olfactory response really occurs at the periphery. In such modulation, behaviorally highly relevant odor information, serving specific purposes such as the detection of pheromones or danger signals, are processed differently. However, whether the differences observed at the OSN level are still apparent in the AL, or whether “signal normalization” (maybe through LNs) ensuring a consistent output occurs is a very interesting question that remains to be investigated.

Furthermore, OSNs of *Drosophila* rely primarily on two types of olfactory receptors, odorant receptors and ionotropic receptors. In the present study, we focused on ORs. However, recent investigations have shown that IRs colocalize more widely with ORs than previously thought [75,76]. Whether IRs influence the OR response was outside the scope of our study, nevertheless it is an interesting possibility that prompts further investigation. In addition, differences in receptor sensitivity observed throughout this study could be due to the dwelling time and the distance between the receptors at which they are expressed in the membrane. Studying the distribution of ORs along the neurons will also contribute to understanding the differences in OR performance.

## Figures and Tables

**Table 1:** Sensitization results for SSR experiments. A significant difference between the first and second AUC indicates sensitization. Data represent mean ± SEM.

Sensillum	Area under the Curve (AUC, f_norm_ · s)		Paired Test		
	1st AUC	2nd AUC	*n*	*t*-test	Wilcoxon
ab3A (Or22a)	3.04 ± 0.35	4.78 ± 0.69	13	* *p* < 0.05	
ab3B (Or85b)	2.61 ± 0.19	3.35 ± 0.33	11	* *p* < 0.05	
ab5A (Or82a)	3.06 ± 0.29	3.85 ± 0.37	17	** *p* < 0.01	
ab5B (Or47b)	2.50 ± 0.09	3.06 ± 0.18	19		** *p* < 0.01
ab4A (Or7a)	2.64 ± 0.26	2.17 ± 0.27	5	ns	
ab4B (Or56a)	3.66 ± 0.27	3.62 ± 0.34	22	ns	
at1 (Or67d)	5.25 ± 0.87	4.79 ± 1.11	14		ns

**Table 2:** Results for response intensity (as the average increase in fluorescence ∆*F*/*F*_0_) after application of the OR ligands. Green indicates neuronal compartments of Or22a-expressing neurons, and red of Or56a-expressing neurons. Data is expressed as mean ± SEM.

	Control		Auranofin	
Response intensity	1st response	2nd response	1st response	2nd response
Outer dendrites	48.88 ± 20.38	71.34 ± 22.23	25.66 ± 9.87	9.55 ± 5.53
Inner dendrites	66.86 ± 13.91	103.8 ± 23.65	46.28 ± 9.18	15.99 ± 6.52
Soma	32.18 ± 8.18	50.47 ± 12.06	29.94 ± 5.55	21.17 ± 7.12
Outer dendrites	64.74 ± 15.36	34.89 ± 4.75	46.03 ± 11.3	14.15 ± 6.24
Inner dendrites	50.79 ± 14.12	35.79 ± 7.92	30.95 ± 10.72	21.95 ± 6.67
Soma	66.02 ± 16	45.14 ± 10.68	39.13 ± 7.5	23.13 ± 7.53

**Table 3:** Fly lines in use.

	Genotype
1	Canton-S (WT)
2	w; UAS-GCaMP6f; Or22a-Gal4
3	w; UAS-GCaMP6f; Or56a-Gal4/TM6B
4	CyO/BL; Or22a-Gal4, UAS-DenMark
5	CyO/BL; Or22a-Gal4, UAS-MitoGFP
6	(CyO)/+; Or56a-Gal4, UAS-DenMark
7	(CyO)/+; Or56a-Gal4, UAS-MitoGFP/TM6B

**Table 4:** Summary of target sensillum and chemicals used for the SSR experiments.

Sensillum Type (Neuron)	Compound	CAS
ab3 (A)	ethyl hexanoate	123-66-0
ab3 (B)	2-heptanone	110-43-0
ab4 (A)	E2-hexanal	6728-26-3
ab4 (B)	geosmin	16423-19-1
at1	cVA	6186-98-7
ab5 (A)	geranyl acetate	105-87-3
ab5 (B)	pentyl acetate	628-63-7

## Data Availability

The data presented in this study are available on request from the corresponding author.

## Supplementary Materials

The following supporting information can be downloaded at: https://www.mdpi.com/article/10.3390/ijms25137125/s1.
