# Supplementary material for: Response Plasticity of Drosophila Olfactory Sensory Neurons
Source: Int J Mol Sci. 2024 Jun 28;25(13):7125. doi: 10.3390/ijms25137125 (PMC11241008; doi:10.3390/ijms25137125)
Supplement: Supplementary file 1 [file ijms-25-07125-s001.zip › ijms-3017327-supplementary.pdf]

Response Plasticity of *Drosophila* Olfactory Sensory Neurons

Lorena Halty-deLeon, Venkatesh Pal Mahadevan, Eric Wiesel, Bill S. Hansson † and Dieter Wicher *,†

Max Planck Institute for Chemical Ecology, 07745 Jena, Germany;
vmahadevan@ice.mpg.de (V.P.M.);
ewiesel@ice.mpg.de (E.W.); hansson@ice.mpg.de (B.S.H.)
* Correspondence: dwicher@ice.mpg.de
† These authors contributed equally to this work.

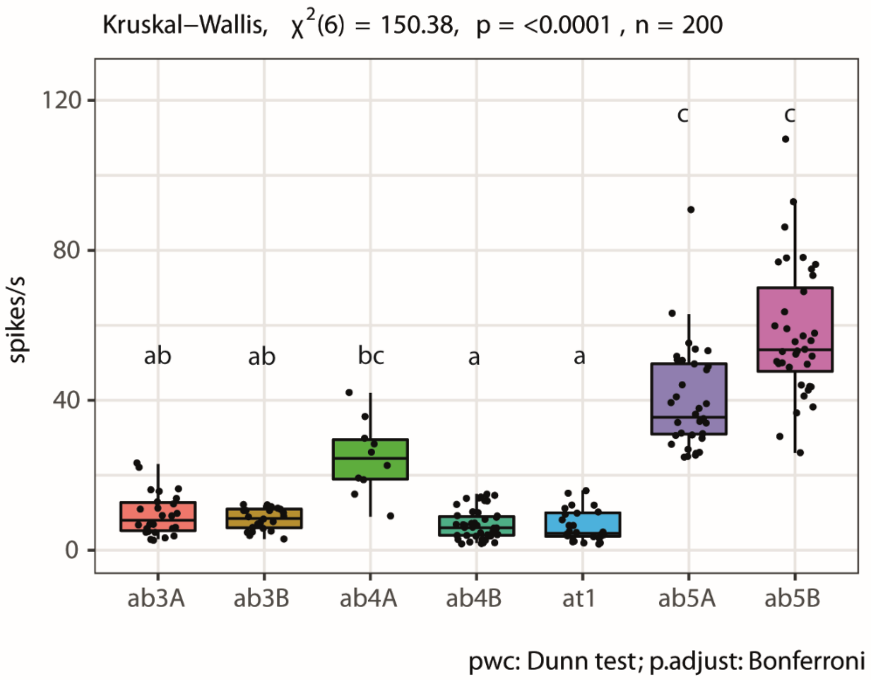

Figure S1. **Spontaneous activity**. Box plots showing the spontaneous activity per second of the different neurons. Different small letters above the boxplots indicate significant differences, $p < 0.05$ (Kruskal-Wallis test followed by Dunn's pairwise (pwc) multiple comparison test). Data represent mean ± SEM. For detailed statistics, see Table S1.

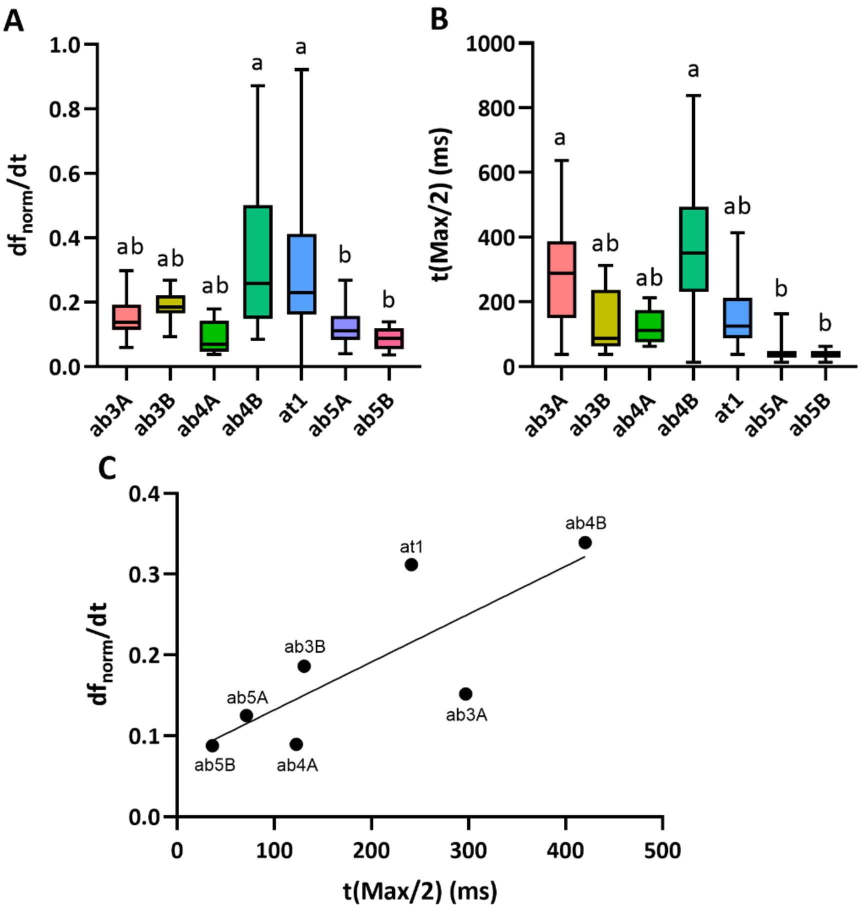

Figure S2. **Response velocity**. **A,B:** Box plots depict the maximum response velocity (**A**) and time until half maximal response velocity (**B**) for each neuron type. Data extracted for **A** as the first derivative (df_{norm}/dt) from curves in Figure 1B. Different small letters above the boxplots indicate significant differences, $p < 0.05$ (Kruskal-Wallis test followed by Dunn's pairwise (pwc) multiple comparison test). Data represent maximum ± SEM, n = 6-22. **C:** Correlation between maximum response velocity and time to half maximal velocity. Spearman correlation, r = 0,8571, p = 0,0238

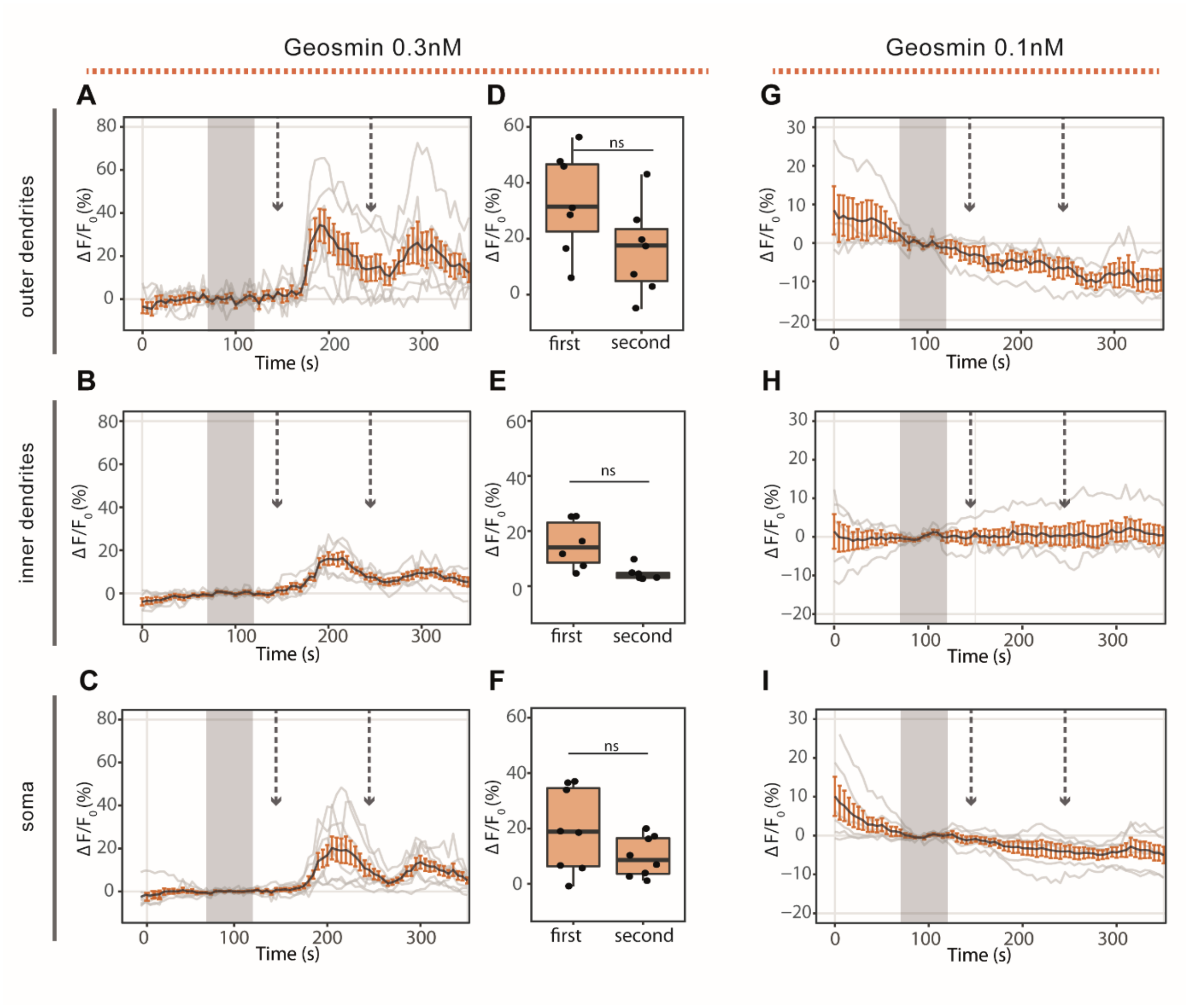

Figure S3. **No sensitization in Or56a neurons**. **A,B,C**: Kinetics show averaged time course of the change in fluorescence intensity (ΔF/F0) in *Drosophila* OSNs after application of 0.3 nM geosmin (arrows). At 0.3 nM there is no difference between applications in outer dendrites (**A**, n=7), inner dendrites (**B**, n=6) and soma (**C**, n=8). **D,E,F**: maximum increase in ΔF/F0 after geosmin application in the different compartments as in A-C. **G,H,I:** No responses are observed after 0.1 nM geosmin application (arrows). Gray bar indicates where data was normalized to obtain ΔF/F0. Data represent mean ± SEM; two-tail paired t-test, ns not significant.

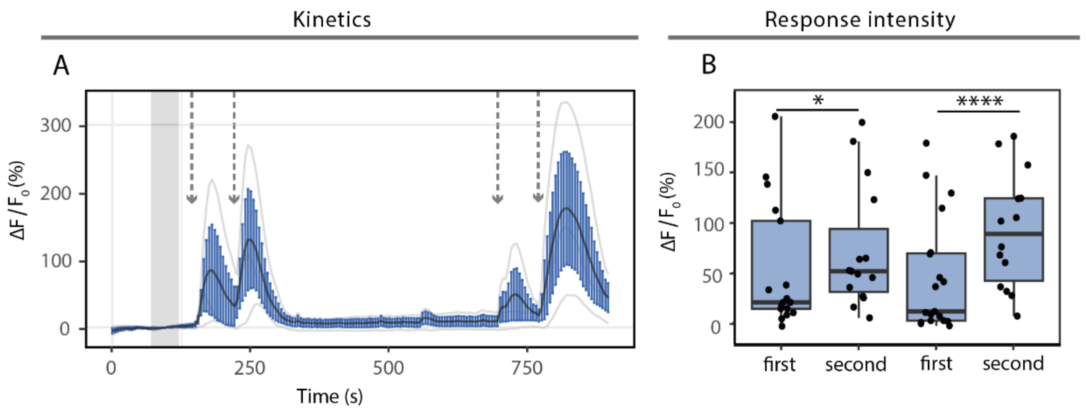

Figure S4. **Standard sensitization induced by Orco Agonist VUAA1**. **A**: Kinetics show averaged time course of the change in fluorescence intensity (ΔF/F0) in *Drosophila* OSNs after application of 30 µM VUAA1 (arrows). Fluorescence intensities were taken from combination of all compartments (n=22). **B**: maximum increase in ΔF/F0 after VUAA1 application. Gray bar indicates where data was normalized to obtain ΔF/F0. Data represent mean ± SEM; Dunn's pairwise multiple comparison test, * $p < 0.05$, **** $p < 0.001$.

Table S1. Spontaneous activity results from Dunn´s multiple comparison test with Bonferroni correction following a Kruskal-Wallis test. Spikes/s show mean + SEM

Group1	Group2	n_1	Spikes/s	n_2	Spikes/s	statistic	p.adj	p.adj.signif
ab3A	ab3B	26	9.58 ± 1	22	8.32 ± 0.6	-0.11052	1	ns
ab3A	ab4A	26	9.58 ± 1	10	24.70 ± 3.1	2.472702	0.281601	ns
ab3A	ab4B	26	9.58 ± 1	44	6.84 ± 0.6	-1.29403	1	ns
ab3A	ab5A	26	9.58 ± 1	34	40.06 ± 2.38	5.245598	3.27E-06	****
ab3A	ab5B	26	9.58 ± 1	36	57.95 ± 2.9	6.853514	1.51E-10	****
ab3A	at1	26	9.58 ± 1	28	6.32 ± 078	-1.52926	1	ns
ab3B	ab4A	22	8.32 ± 0.6	10	24.70 ± 3.1	2.496476	0.263412	ns
ab3B	ab4B	22	8.32 ± 0.6	44	6.84 ± 0.6	-1.10326	1	ns
ab3B	ab5A	22	8.32 ± 0.6	34	40.06 ± 2.38	5.111615	6.71E-06	****
ab3B	ab5B	22	8.32 ± 0.6	36	57.95 ± 2.9	6.636389	6.75E-10	****
ab3B	at1	22	8.32 ± 0.6	28	6.32 ± 078	-1.34952	1	ns
ab4A	ab4B	10	24.70 ± 3.1	44	6.84 ± 0.6	-3.54014	0.008398	**
ab4A	ab5A	10	24.70 ± 3.1	34	40.06 ± 2.38	1.241198	1	ns
ab4A	ab5B	10	24.70 ± 3.1	36	57.95 ± 2.9	2.360504	0.383253	ns
ab4A	at1	10	24.70 ± 3.1	28	6.32 ± 078	-3.62818	0.005994	**
ab4B	ab5A	44	6.84 ± 0.6	34	40.06 ± 2.38	7.386824	3.16E-12	****
ab4B	ab5B	44	6.84 ± 0.6	36	57.95 ± 2.9	9.273146	3.80E-19	****
ab4B	at1	44	6.84 ± 0.6	28	6.32 ± 078	-0.39877	1	ns
ab5A	ab5B	34	40.06 ± 2.38	36	57.95 ± 2.9	1.66126	1	ns
ab5A	at1	34	40.06 ± 2.38	28	6.32 ± 078	-6.98715	5.89E-11	****
ab5B	at1	36	57.95 ± 2.9	28	6.32 ± 078	-8.65314	1.05E-16	****
